# Efforts of the human immune system to maintain the peripheral CD8+ T cell compartment after childhood thymectomy

**DOI:** 10.1186/s12979-016-0058-z

**Published:** 2016-02-02

**Authors:** Manuela Zlamy, Giovanni Almanzar, Walther Parson, Christian Schmidt, Johannes Leierer, Birgit Weinberger, Verena Jeller, Karin Unsinn, Matthias Eyrich, Reinhard Würzner, Martina Prelog

**Affiliations:** Department of Pediatrics, Medical University Innsbruck, Innsbruck, Austria; Department of Pediatrics, University Hospital Wuerzburg, University of Wuerzburg, Josef-Schneider-Str. 2, 97080 Wuerzburg, Germany; Institute of Legal Medicine, Medical University Innsbruck, Innsbruck, Austria; Penn State Eberly College of Science, University Park, PA USA; Department of Haematology and Oncology, University of Greifswald, Greifswald, Germany; Department of Internal Medicine, Medical University Innsbruck, Innsbruck, Austria; Institute for Biomedical Aging Research, University of Innsbruck, Innsbruck, Austria; Department of Radiology, Medical University Innsbruck, Innsbruck, Austria; Department of Hygiene and Medical Microbiology, Medical University Innsbruck, Innsbruck, Austria

**Keywords:** Thymectomy, Naive T cells, CD8, TRECs, Telomeres, TCR diversity, CMV

## Abstract

**Background:**

Homeostatic mechanisms to maintain the T cell compartment diversity indicate an ongoing process of thymic activity and peripheral T cell renewal during human life. These processes are expected to be accelerated after childhood thymectomy and by the influence of cytomegalovirus (CMV) inducing a prematurely aged immune system.

The study aimed to investigate proportional changes and replicative history of CD8+ T cells, of recent thymic emigrants (RTEs) and CD103+ T cells (mostly gut-experienced) and the role of Interleukin-(IL)-7 and IL-7 receptor (CD127)-expressing T cells in thymectomized patients compared to young and old healthy controls.

**Results:**

Decreased proportions of naive and CD31 + CD8+ T cells were demonstrated after thymectomy, with higher proliferative activity of CD127-expressing T cells and significantly shorter relative telomere lengths (RTLs) and lower T cell receptor excision circles (TRECs). Increased circulating CD103+ T cells and a skewed T cell receptor (TCR) repertoire were found after thymectomy similar to elderly persons. Naive T cells were influenced by age at thymectomy and further decreased by CMV.

**Conclusions:**

After childhood thymectomy, the immune system demonstrated constant efforts of the peripheral CD8+ T cell compartment to maintain homeostasis. Supposedly it tries to fill the void of RTEs by peripheral T cell proliferation, by at least partly IL-7-mediated mechanisms and by proportional increase of circulating CD103+ T cells, reminiscent of immune aging in elderly. Although other findings were less significant compared to healthy elderly, early thymectomy demonstrated immunological alterations of CD8+ T cells which mimic features of premature immunosenescence in humans.

## Background

The thymus plays an essential role in the differentiation of T cells, which are necessary for an effective cellular immune response against pathogens and tumor cells and for control of self-reactive T cell clones. TCR rearrangement within the thymus generates the basis for a wide TCR repertoire. The thymus is fully developed at birth and reaches its largest size during childhood with subsequent structural changes. The decline of *de novo* T cell production accelerates with puberty with a decreasing rate of approximately 3 % per year during adulthood [1]. Although proportionally declining with age, the number of naive T cells is maintained by peripheral proliferation of pre-existing naive T cells which results in a dilution of T cell receptor excision circles (TRECs) within thymus-derived naive T cells [2–4] and in shortening of the relative telomere lengths (RTLs) by increased replication rounds [5]. IL-7 is known as an essential factor involved in maintenance of the peripheral naive T cell pool, in regulation of T cell homeostasis and in preservation of the TCR repertoire [6]. IL-7 may also participate in the reconstitution of peripheral T cell subpopulations in conditions of low thymic output [7, 8].

In patients who were partly or totally thymectomized in early childhood due to surgery for congenital heart defects [1, 9, 10], several studies have revealed multiple immune alterations within the peripheral T cell compartments [11–21] and a delayed humoral immune response to new antigens later in life [22, 23]. Cytomegalovirus (CMV) is known to drive the T cell differentiation towards abundance of terminally differentiated CD28- effector T cells and towards a restricted TCR repertoire [24] which was also seen in a subgroup of young adults thymectomized during early childhood (YATEC) similar to elderly individuals [17]. These exacerbated alterations were seen as the likely consequence of the chronic stimulation of the T cell immune system caused by the life-long persistence of CMV in the absence of an adequate T cell renewal capacity [1, 17].

The present study aimed to perform an in-depth analysis of proportional changes of CD8+ T cell subpopulations with inclusion of recent thymic emigrants (RTE) [25, 26] and gut-experienced CD103+ T cells [27]. The role of IL-7 and IL-7 receptor (CD127)-expressing T cells, as well as the proportion of cells that are outside the G0 stage at the time point of blood withdrawal (Ki67 expression) and replicative history of peripheral CD8+ T cells by TRECs and RTLs were studied in order to assess possible mechanisms of maintenance of the peripheral naive T cell compartment under lack of sufficient thymic output as expected in thymectomized individuals. Differentiation of CD8+ T cells and TCR diversity were investigated under the light of peripheral T cell exhaustion by chronic stimulation caused by CMV which is known to influence a prematurely aged immune system and was thought to underline the hypothesis of premature T cell immunosenscence in thymectomized humans. We could demonstrate that immunological alterations associated with thymectomy particularly affected the CD8+ T cell pool.

## Methods

### Study population

Peripheral blood mononuclear cells (PBMCs) were collected from young adults or adolescents thymectomized in early childhood at ≤24 months of age (YATEC), from young adults or adolescents thymectomized in childhood at >24 months of age (YAT), from young healthy controls (YHC) as a control group for YATEC and YAT and from older healthy controls (OHC) aged >65 years as a control group for immunosenescence parameters (Table 1). Thymectomy was performed during open heart surgery by total resection of both lobes for surgical reasons with inclusion and exclusion criteria described in detail previously [16]. Reconstitution of the thymus was excluded by magnetic resonance imaging. The study was performed according to the Declaration of Helsinki with approval by the local Ethics Committee, Medical University Innsbruck. All participants or their legal representatives gave written informed consent.

**Table 1:** Demographics of study populations and proportions of lymphocytes

	YATEC	YAT	YHC	OHC
Number (female/male)	23 (5/18)	12 (7/5)	17 (11/6)	9 (4/5)
CMV (positive/negative)^a^	10/13	3/8	5/12	5/2
Age (years)^b^	19.7 ± 8.1 (17.9; 9.0–35.8)	18.4 ± 7.2 (17.4; 9.2–31.3)	23.5 ± 7.9 (25.3; 8.0–33.0)	72.8 ± 4.4 (73.4; 67.0–80.0)
Age at thymectomy (years)^b^	0.5 ± 0.5 (0.2; 0.01–1.7)	7.6 ± 5.0 (5.1; 2.1–16.8)	n. a.	n. a
Time post thymectomy (years)^b^	19.7 ± 7.6 (19.7; 8.8–34.2)^c^	14.4 ± 6.1 (14.6; 3.9–26.1)^c^	n. a.	n. a.
Lymphocytes absolute/μl^b^	1.8 ± 0.5 (1.7; 1.3–2.7)	2.1 ± 0.5 (2.1; 1.7–2.5)	2.0 ± 0.4 (2.1; 1.3–2.7)	1.6 ± 0.7 (1.3; 0.9–2.7)
CD3+ (% of lymphocytes)^b^	70.8 ± 11.9 (75.0; 48.0–85.0)	79.2 ± 2.2 (77.9; 78.0–82.0)	79.0 ± 7.6 (80.9; 62.0–89.0)	69.8 ± 11.8 (70.8; 50.0–85.0)
CD4+ (% of lymphocytes)^b^	44.6 ± 13.7 (43.0; 35.1–61.4)	51.5 ± 6.8 (48.5; 46.7–59.3)	51.8 ± 6.6 (54.2; 36.5–61.6)	43.2 ± 12.2 (43.6; 28.5–65.9)
CD8+ (% of lymphocytes)^b^	24.7 ± 7.5 (22.3; 15.4–39.9)	25.7 ± 7.1 (26.3; 16.3–32.4)	25.8 ± 6.9 (26.0; 12.3–38.2)	25.2 ± 8.2 (23.4; 14.3–39.0)

*Abbreviations*: young adults/adolescents thymectomized in early childhood at ≤24 months of age, *YATEC*; young adults/adolescents thymectomized in childhood at >24 months of age, *YAT*; young healthy controls, *YHC*; old healthy controls, *OHC*; not applicable, n. a

^a^CMV serology was unknown in 2 cases of the OHC

^b^Values are given in mean ± standard deviation (median; range)

^c^Chronological age significantly correlated with time post thymectomy in YATEC (R^2^–0.958; *p* = 0.0001) and in YAT (R^2^ = 0.853; *p* = 0.0001)

### Definition and quantification of T cell subpopulations

PBMCs were incubated with fluorochrome-labeled monoclonal antibodies (mAbs) (BD Pharmingen, San Jose, CA, USA) and analyzed by FACS Calibur flow cytometer (Becton Dickinson, Oxford, United Kingdom) and CELLQuest software (BD Pharmingen), as described previously [16]. A minimum of 3,000 events was counted for each panel in FACS analysis with results expressed as percentages of gated lymphocytes. For technical limitations regarding available blood volumes, subgroup analysis could not be performed in all subjects. CD45RA, CD27 and CCR7 were used to differentiate between naive (CD45RA + CD27 + CCR7+), early memory (CD45RA-CD27 + CCR7+), late memory (CD45RA-CD27-CCR7-) and terminally differentiated effector (CD45RA + CD27-CCR7-) T cells re-expressing CD45RA [28]. CD31 was previously used to identify RTE in CD4+ T cells [26]. For CD8+ RTEs, CD31 is less well established, but was used as a naive CD8+ T cell marker [25]. CD127, the IL-7 receptor α chain, is generally expressed on T cells susceptible to auto-proliferative mechanisms by IL-7, and, thus, was used mainly in combination with naive T cell markers [28]. Ki67 was used to label the proportion of cells that are outside the G0 stage of their existence [16, 18], reflecting the immune activation status of the individuals at the time point of blood withdrawal. CD103 has been described as a marker of mucosa-derived T cells [19, 27] and of CD8+ T cells which have entered the gut [29].

### ELISA tests

Serum IgG directed against CMV (Enzygnost, Dade Behring, Vienna, Austria) and serum IL-7 concentrations were measured by ELISA (BD Pharmingen, San Jose, CA, USA) according to standard laboratory methods.

### TCR spectratyping

In order to analyze the clonal composition of the TCR repertoire total RNA was extracted from PBMCs and reverse-transcribed. TCR Vβ transcripts were amplified by PCR using different primers for each of the 24 Vβ families and a specific primer for the constant region of the β chain labeled with the fluorescent dye marker 6-FAM [30]. Aliquots of the PCR product were analyzed on CE 3100 Genetic Analyzer (Perkin Elmer, Norwalk, CT). Raw data were analyzed using GeneScan 3.7 and Gentyper 3.6 software packages (Applied Biosystems, Foster City, CA) using the Local Southern method for fragment size estimation [31]. Scores for clonality were assigned based on the occurrence of dominant clonal expansions for each Vβ family. Clonality score 1 was used for peaks showing Gaussian distribution, 2 for several peaks and 3 for one peak, as described previously [32, 33].

### Quantification of TRECs and relative telomere length

DNA was extracted from separated CD8 + CD45RA+ T cells after magnetic activated cell sorting (MACS) (Milteny Biotec, Bergisch-Gladbach, Germany) using QIAamp DNA Mini Kit (Qiagen, Chatsworth, CA, USA) as described previously [22]. Signal-joint TREC concentrations were determined by real-time PCR as described in detail previously [26, 34]. To avoid bias by different numbers of naive T cells, TRECs were calculated in relation to CD8 + CD45RA+ T cell numbers [3]. Determination of relative telomere length (RTL) was performed by calculating the ratio of a quantitative PCR reaction product from the same sample using specific primers for telomeres and a single copy gene as described previously [35–37]. In absence of DNA samples from our OHC cohort, TRECs and telomeres were analyzed in samples of leucocytes from 10 healthy donors (5 female, 5 male) aged 71 to 78 years as an internal control for aged individuals and measured with the method described above.

### Statistical analysis

Shapiro-Wilks test was used to test for normal distribution. Non-parametric Mann–Whitney-U was applied for not normally-distributed independent variables. To avoid bias by multiple testing, a *p*-value ≤0.05 was considered statistically significant using the less conservative Benjamini-Hochberg-correction to reduce false-positive results by the following assumption: A *p*-value was considered statistically significant in the case that a *p*-value was below the highest *p*-value fulfilling the following requirement that p(i) ≤ (i*q)/m. An arbitrarily set q-value indicates the tolerance for false-significant results (in this case q = 0.1), with i indicating the rank in a step-up ranking of *p*-values and m indicating the total number of executed tests. *X*^2^ test was used to compare dichotome variables. Correlations between variables were identified by Spearman’s rank correlation coefficient. A generalized linear model was generated by step-to-step regression to infer the influence of the time post thymectomy, the age at thymectomy or CMV positivity on the immunological system, adjusted for the chronological age (age at blood withdrawal) of the patient. All statistical analyses were performed with SPSS Version 22.0 (Chicago, IL, USA).

## Results

### Lower proportions of naive CD8+ T cells after thymectomy

**Fig. 1:**
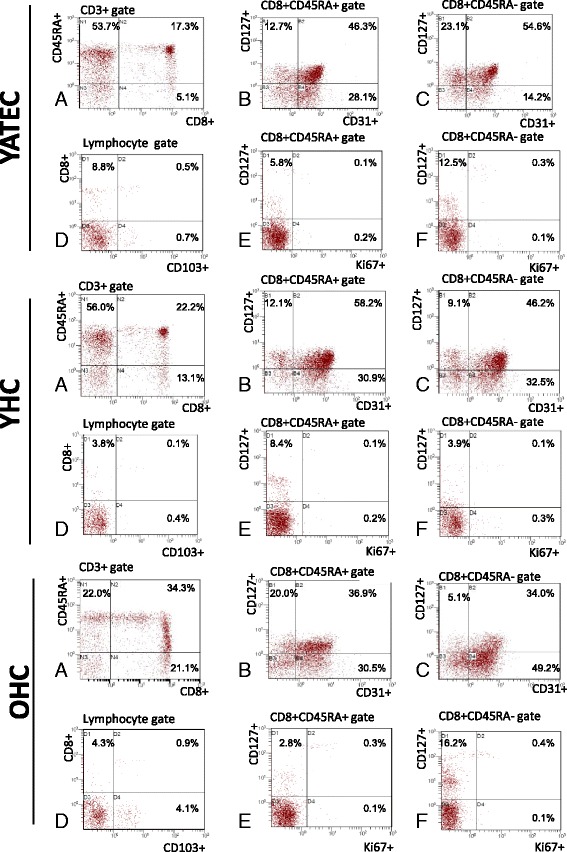
Analysis of CD127+, CD31+ and CD103+ CD8+ T cell proportions. Representative examples of flow cytometric analysis of CD8+ T cells of one YATEC patient, one YHC and one OHC are shown. Gating strategies were as follows: First, CD8+ T cells were analyzed within the CD3+ lymphocyte gate. Percentages of CD45RA+ and CD45RA- were analyzed in CD8+ within the CD3+ T cell gate (**a**). CD127+ was determined within CD45RA+ (**b**) and CD45RA- CD8+ T cells (**c**) together with CD31, percentages indicating positive events in the CD45RA+ or CD45RA- CD8+ T cell gate, respectively. Due to low percentages of circulating CD103 + CD8+, these cells were determined in the total lymphocyte gate (**d**). Percentages of Ki67+ were determined together with CD127+ in the CD45RA+ (**e**) and CD45RA- CD8+ gate (**f**), respectively

**Fig. 2:**
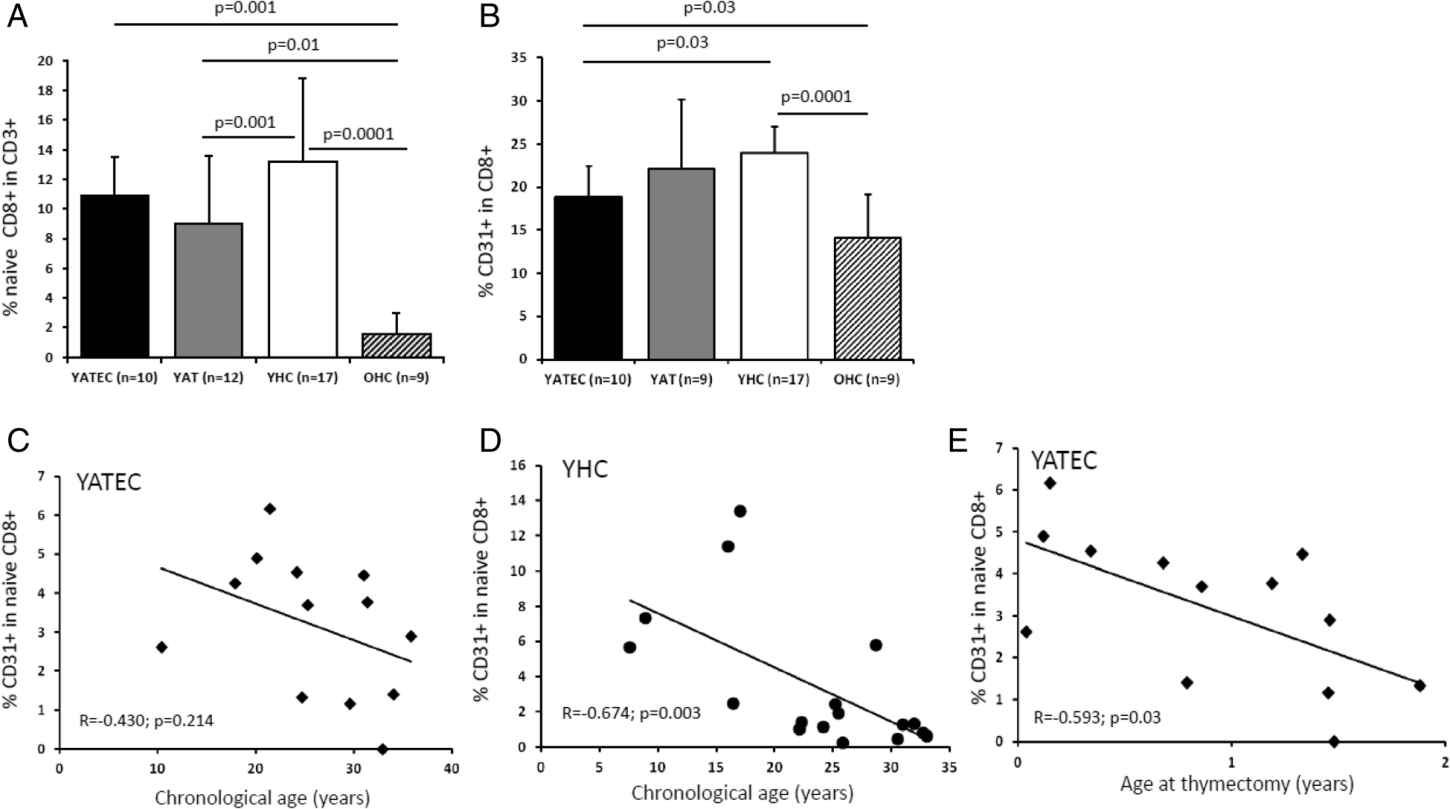
Proportions of naive and CD31+ CD8+ T cells. YAT patients showed lower proportions of naive CD8+ T cells than YHC (**a**). YATEC patients demonstrated lower proportions of CD31 + CD8+ T cells than YHC. Lowest naive and CD31+ CD8+ T cells were found in OHC compared to YHC (**b**). Bars represent mean percentages ± standard deviation, numbers (n) of investigated individuals for each group. A p ≤ 0.05 indicates statistical significance (Mann–Whitney *U* test). YHC showed a negative correlation of CD31+ naive CD8+ T cells with chronological age (**d**), whereas YATEC did not (**c**). In YATEC patients, CD31+ naive CD8 + T cells negatively correlated with age at thymectomy (**e**). Spearman’s Rank correlation coefficient, R; *p* ≤ 0.05 indicates statistical significance

By guiding cells to and within lymphoid organs, CCR7 contributes to immunity and tolerance and is a characteristic marker of naive, regulatory and memory T cells [38]. Together with CD27 it is used to define peripheral naive T cells [28]. YAT showed significantly lower proportions of naive CD8+ T cells compared to YHC (Fig. 1, Fig. 2a).

### Lower proportions of CD31-expressing CD8+ T cells after thymectomy

CD31 has been reported as characteristic marker of RTE decreasing with age [26, 39]. For the purpose to evaluate the peripheral existence of CD31+ T cells in the condition of expected low production of RTE in thymectomized patients, we assessed the proportions of CD31-expressing T cells within the CD8+ T cell pool. Significant lower proportions of CD31+ in CD8+ T cells were found in YATEC compared to YHC (Fig. 1, Fig. 2b). In contrast to YATEC, only YHC showed a negative correlation of CD31+ naive CD8+ T cells with chronological age (Fig. 2c, d). In YATEC patients, CD31+ naive CD8 + T cells negatively correlated with age at thymectomy, with a trend towards lower proportions when being thymectomized in the second year of life (Fig.2e). This finding was also confirmed by linear regression (R^2^ = 0.445) including chronological age, CMV and age at thymectomy, which revealed age at thymectomy as an independent factor for reduction of CD31+ naive CD8 + T cells (*p* = 0.04).

### Increased Ki67+ expression of CD127-expressing T cells and lower IL-7 serum concentrations after thymectomy

**Fig. 3:**
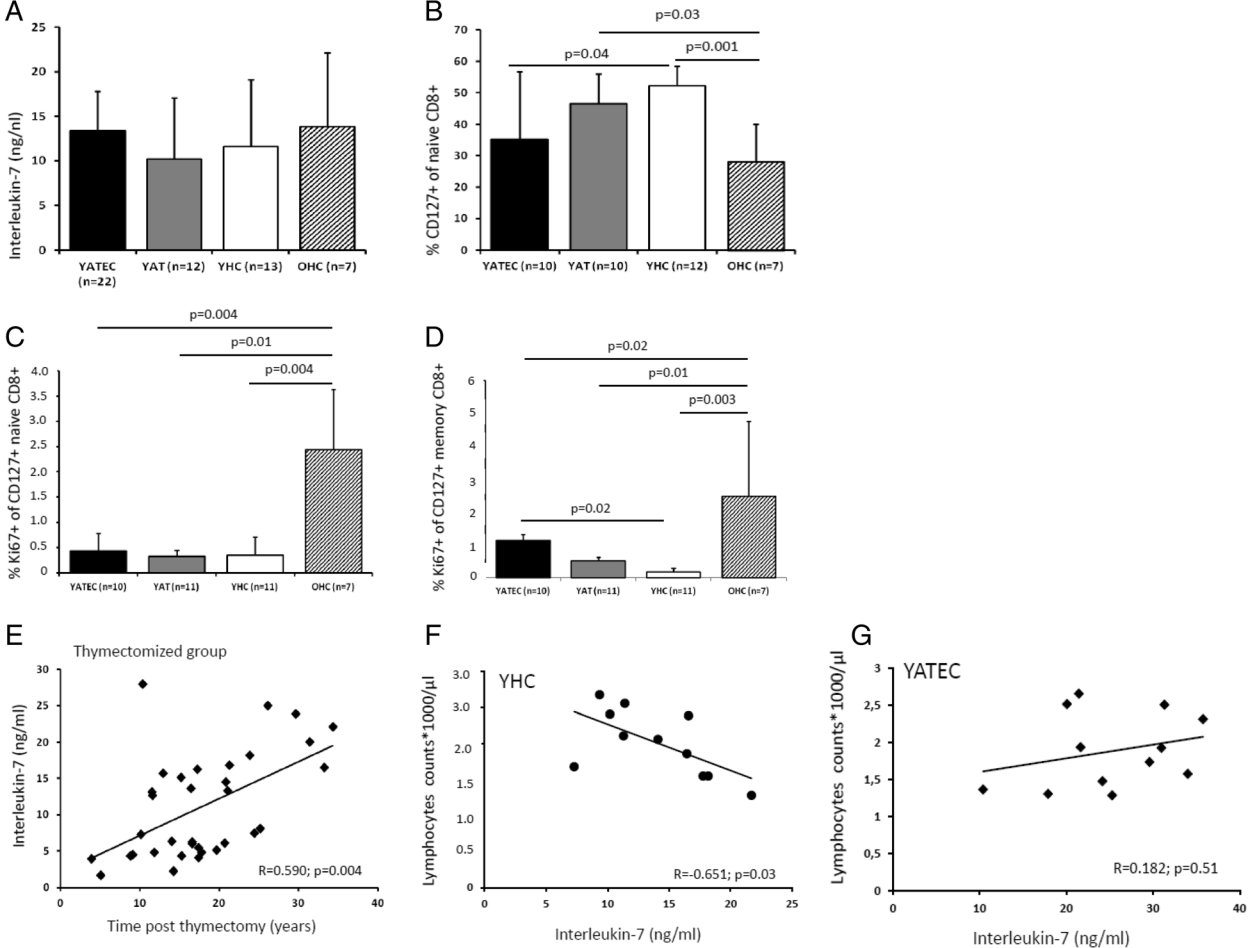
Serum Interleukin-7 concentrations, proportions of CD127+ and Ki67+ CD8+ T cells. Serum IL-7 concentrations are shown in YATEC and YAT patients compared to YHC and OHC (**a**). Lower proportions of CD127+ naive CD8+ T cells were seen in YATEC patients compared to YHC, with lowest proportions in OHC (**b**). Ki67-expressing cells were demonstrated within the CD127+ naive (**c**) and memory CD8+ T cell subpopulations (**d**). Highest percentages of Ki67+ were demonstrated in OHC. Bars represent mean percentages ± standard deviation, numbers (n) of investigated individuals for each group. A *p ≤* 0.05 indicates statistical significance (Mann–Whitney *U* test). Taken together, in YATEC and YAT (“thymectomized group”) serum Interleukin-7 (IL-7) concentrations showed a significant correlation with time post thymectomy (**e**). Lymphocyte counts significantly correlated with IL-7 concentrations in YHC (**f**), whereas YATEC did not (**g**). Spearman’s Rank correlation coefficient, R; *p* ≤ 0.05 indicates statistical significance

We next aimed to study the potential role of IL-7 and CD127-expressing T cells in their contribution to the peripheral naive T cell compartment in thymectomized individuals (Fig. 3a). IL-7 concentrations positively correlated with time post thymectomy in YATEC (Fig. 3e). YATEC thymectomized more than 25 years ago and over 30 years of age (YATEC > 30a) showed a trend towards higher IL-7 concentrations compared to YHC > 30a (Fig. 5c). As higher concentrations of IL-7 were known from lymphopenic conditions [7, 8], IL-7 concentrations were set in relation to lymphocyte counts. IL-7 concentrations positively correlated with lymphocyte counts only in YHC but not in YATEC (Fig. 3f, g).

For further investigation of naive CD8+ T cells, CD127 was included into the analysis due to its function as IL-7 receptor α chain. A trend to lower proportions of CD127+ cells within naive CD8+ T cells were seen in YATEC and YAT compared to YHC (Fig. 1, Fig. 3b).

In search for the proliferative activation of peripheral CD45RA+ naive or CD45RA- memory CD8+ T cells expressing CD127+ and, thus, expected to be susceptible to IL-7 activity, higher Ki67-expression was found in CD127+ memory CD8+ T cells in YATEC compared to YHC but not in CD127+ naive CD8+ T cells (Fig. 1, Fig. 3c, d). No correlations were seen between IL-7 concentrations, proportions of CD127-expressing T cells and Ki67 expression in any groups.

### Replicative history of naive T cells

**Fig. 4:**
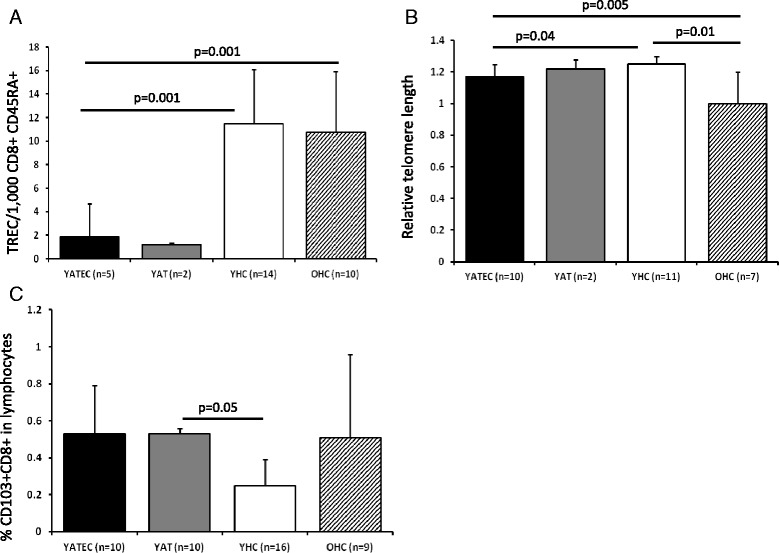
Replicative history of naive T cells. Significantly lower TRECs per 1,000 CD8 + CD45RA+ T cells (**a**) and shorter relative telomere lengths (RTLs) (**b**) were found in YATEC patients compared to YHC. Bars represent mean percentages ± standard deviation, numbers (n) of investigated individuals for each group. No statistical analysis was performed with YAT, as only 2 of them had detectable TRECs and RTLs. Higher proportions of CD103-expressing lymphocytes in CD8 + CD103+ T cells were seen in YATEC patients compared to YHC (**c**). A *p* ≤ 0.05 indicates statistical significance (Mann–Whitney *U* test)

**Fig. 5:**
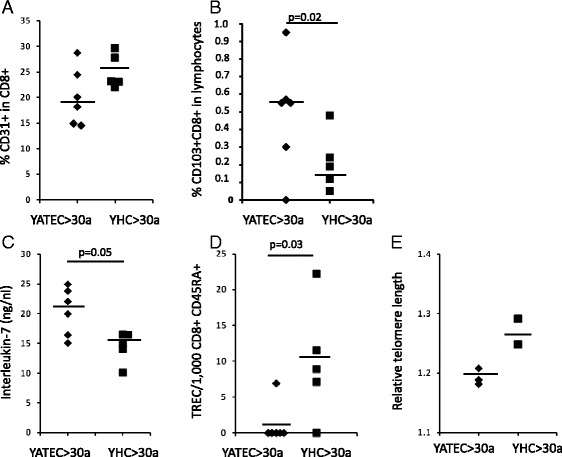
Proportions of CD31+ and CD103+ CD8+ T cells, Interleukin-7 (IL-7) concentrations, TRECs and relative telomere lengths in YATEC aged >30 years (YATEC > 30a) and YHC aged >30 years (YHC > 30a). Proportions of CD31+ (**a**) and CD103+ cells in CD8+ T cells (**b**) are shown in YATEC aged >30 years (YATEC > 30a) who had thymectomy more than 25 years ago compared to YHC aged >30 years (YHC > 30a). Significantly higher proportions of CD103+ CD8+ T cells (**b**) and a trend for higher IL-7 concentrations (**c**) were found in YATEC > 30a compared to YHC > 30a. Negative TRECs were seen in five YATEC > 30a compared to YHC > 30a with only one individual with negative TRECs (**d**). A trend to lower relative telomere lengths (RTLs) was demonstrated in YATEC > 30a compared to YHC > 30a (**e**). Horizontal lines indicate the median. A *p* ≤ 0.05 indicates statistical significance (Mann–Whitney *U* test)

TRECs were detectable in only 5 YATEC patients (14.7 %) compared to 14 YHC (82.4 %) (*p* = 0.001). In YATEC, TRECs were significantly lower compared to YHC (Fig. 4a). Telomeres were significantly shorter in YATEC than in YHC (Fig. 4b). These findings were also significant in the comparison between YATEC > 30a and YHC > 30a (Fig. 5d, e).

### Proportions of CD103+ T cells

CD103, an α E integrin, necessary for T cell homing and retention in the gut or other epithelia, is highly expressed in gut-derived or mucosa-experienced T cells, particularly in CD8+ T cells [29, 40]. To search for possible extra-thymic T cell generation or increased homing activity of CD8+ T cells to gut mucosa, expression of CD103 was analyzed in YATEC. Higher proportions of CD103+ CD8+ T cells within the total lymphocyte gate were found in YAT compared to YHC (Fig. 1, Fig. 4c), which could be also shown for YATEC > 30a compared to YHC > 30a (Fig. 5b).

### Influence of CMV on lymphocyte subpopulations

**Fig. 6:**
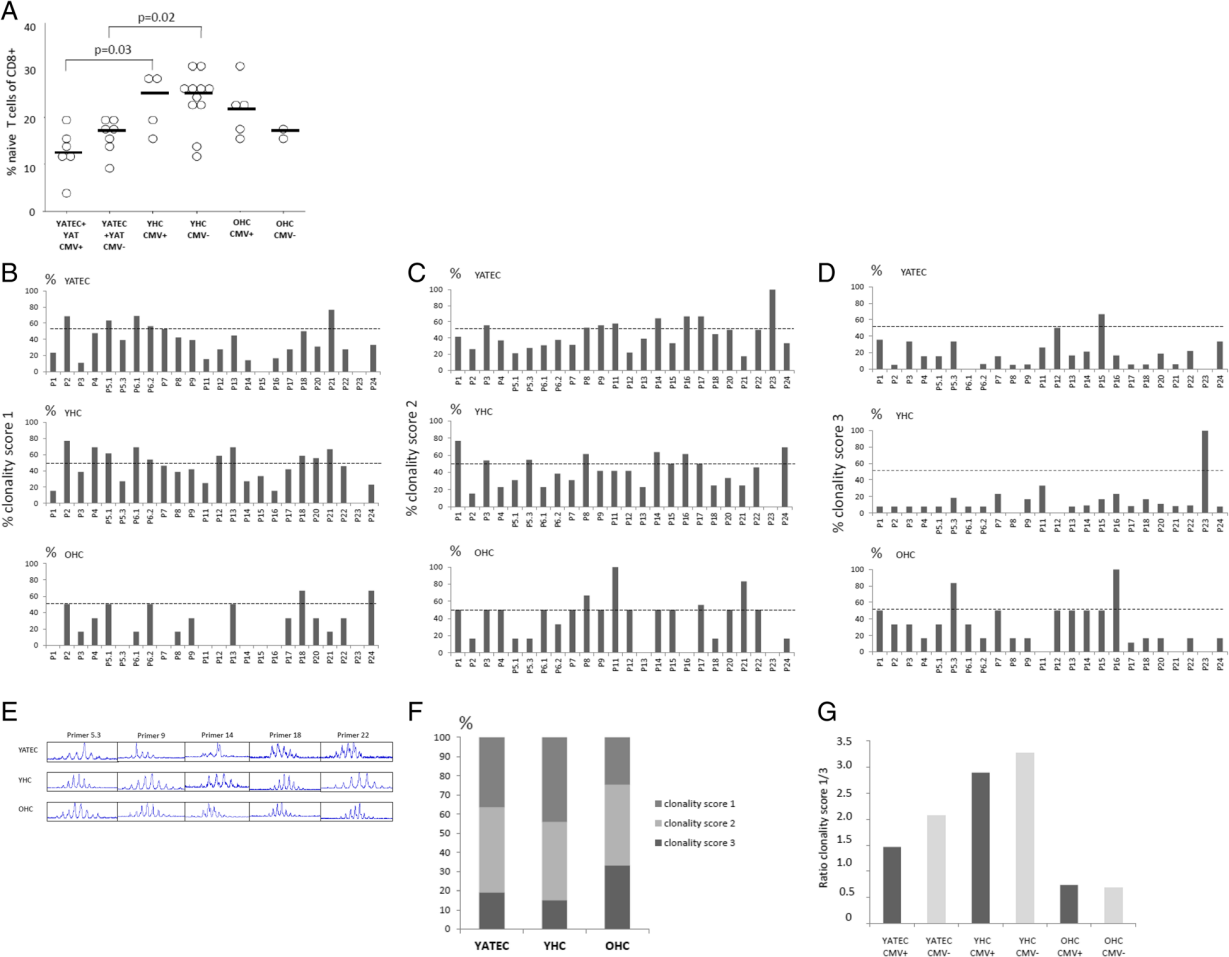
Association between CMV-seropositivity, proportions of naive T cells and TCR Vβ repertoire diversity. No significant differences were seen between CMV positive (CMV+) or negative (CMV-) YATEC patients or YHC regarding proportions of naive CD4+ T cells (**a**). CMV positive YATEC patients had lower proportions of naive CD8+ T cells than CMV positive YHC and CMV negative YATEC (**b**). Horizontal line indicates the median. A *p* ≤ 0.05 indicates statistical significance (Mann–Whitney *U* test). Percentages of clonality score 1 (polyclonal) (**c**), score 2 (oligoclonal) (**d**) and score 3 (monoclonal) (**e**) are given for all evaluated Vβ families for YATEC patients, YHC and OHC. An arbitrary dotted line indicates 50 % reaching clonality score (C-E). Representative repertoire profiles for 5 out of 24 evaluated Vβ family primers are provided for one YATEC patient, one YHC and one OHC showing a skewed TCR repertoire in YATEC and OHC (**f**). More monoclonal (clonality score 3) and less polyclonal distributions (clonality score 1) were found in YATEC patients and OHC compared to YHC (**g**). The polyclonal/monoclonal distribution ratio (ratio clonality score 1/3) was lower in CMV positive YATEC and YHC compared to CMV negative individuals (**h**). Lower ratios were found in YATEC patients compared to YHC, with lowest ratios in OHC (**h**). Data represent a trend of scores, but differences between distributions (polyclonal/monoclonal) and CMV serostatus (positive/negative) did not reach statistical significance in any group (*X* ^2^ test)

CMV may drive T cell differentiation by chronic stimulation [24] and accelerate peripheral T cell exhaustion in the case of low thymic ouput as known from elderly persons [41] and, thus, may influence our results in thymectomized patients. To answer the question whether latent CMV infection may have an impact on proportions of naive T cells in thymectomized patients, groups were separated into CMV positive and negative subgroups. Due to small group size, thymectomized patients were not separated into YATEC and YAT for analysis of associations between CMV positive and negative subgroups. Despite a trend to lower naive CD8+ T cells in CMV positive thymectomized patients, no significant differences between CMV positive and negative individuals were found within each group regarding naive T cells (Fig. 6a), CD31+, early and late memory T cells, CD103+ T cells or effector T cells (data not shown). The proportions of CD127-expressing T cell subpopulations were not affected by CMV positivity. Ki67-expressing T cells were not influenced either (data not shown). Performing multiple regression analysis (R^2^ = 0.622) including chronological age, age at thymectomy and CMV seropositivity, CMV (*p* = 0.009) and age at thymectomy (*p* = 0.035) were significantly influencing factors for reduced proportions of naive CD8+ T cells.

### TCR diversity

Physiological involution of the thymus results in a marked loss of TCR diversity which can be accelerated by CMV positivity [32, 33]. Thus, clonality of the TCR was investigated in YATEC, YHC and OHC by TCR Vβ spectratype analysis (Fig. 6b–g). Mild to strong alterations were seen in diversity compared to healthy controls, with fewer polyclonal distributions among the 24 Vβ gene regions in the YATEC group compared to YHC (Fig. 6f). CMV positivity resulted in higher monoclonal patterns which was more pronounced in YATEC patients but did not reach statistical significance (Fig. 6g).

## Discussion

The present study demonstrated that after childhood thymectomy, the peripheral T cells undergo characteristic proportional alterations, particularly of the CD8+ T cell compartment, with increased CD127 cell surface expression and proliferative activity and accumulation of circulating CD103+ T cells. These changes were interpreted as efforts of the peripheral T cell system to maintain homeostasis under the condition of thymic depletion. Together with these features, a skewed TCR repertoire and lower proportions of naive CD8+ T cells in some CMV positive YATEC patients were reminiscent of findings from aged individuals.

Naive T cells seem to be greatly afflicted by childhood thymectomy, as demonstrated by the present study and shown by our previous studies [16, 22] and others [12, 19]. Proportional changes of naive and CD31-expressing T cells were evident in the CD8+ T cell compartment and lack correlation with age in YATEC patients. The well known almost linearly decrease of CD31+ naive T cells with age was confirmed only in YHC [26, 28, 39, 42–44]. The loss of age correlation in YATEC patients may point to other factors than chronological age influencing naive T cell proportions, such as time post thymectomy, as shown in our previous study by assessment of T cell receptor excision circles [16], age at acquiring chronic viral infections, such as CMV, and age at thymectomy. Proportions of CD31+ naive CD8 + T cells were independently influenced by age at thymectomy with lower proportions in those patients who had thymectomy later. Significantly lower naive CD8+ T cells were also found in YAT compared to YHC. This fits well to the speculation that removal of thymic tissue in the first months of life may have less influence on the alterations of the T cell pool than the long-term effects of thymectomy during later childhood due to higher regenerative potential in younger children [12, 20, 23].

Homeostatic proliferation of naive T cells is closely related to IL-7 concentrations, with CD127 expression being critical in regulating IL-7 functions [20, 45–49]. On separated subsets of human peripheral CD8+ T cells, almost 100 % of naive (CD45RA + CCR7+) and central memory (CD45R0 + CCR7+) T cells and 60–70 % of effector memory (CD45R0 + CCR7-), but only <20 % of terminally differentiated (CD45RA + CCR7-) T cells express CD127 [50–52] which agrees with our results. Higher Ki67 expression was measured in CD127+ memory CD8+ T cells in YATEC patients, indicating also compensatory expansion of CD45R0+ memory phenotype T cells probably to fill the void of CD45RA+ naive T cells, as observed previously by our group [16] and by others [12, 15]. This is in agreement with findings, that IL-7 signaling is not only exclusively responsible for the homeostatic proliferation of naive T cells but also of CD8+ memory T cells [53–55], with CD127+ T cells being the population to survive and to develop into long-lived CD8+ memory T cells [55]. Higher proliferative activity in CD127-expressing T cells and a trend towards higher serum IL-7 concentrations in thymectomized patients with increasing age after thymectomy may indicate a role of IL-7 and its receptor in peripheral T cell renewal after thymectomy. The lack of any correlation between serum IL-7 concentrations, CD127 expression and proliferative activity of peripheral T cells in our cohort may be explained by the findings described by others that responsiveness to IL-7 and IL-7-induced down-regulation of CD127 depends very much on cellular activation and additional stimulatory signals and displays to be a dynamic process [56–60]. Studies reported the regulation of T cell homeostasis by IL-7 and the improvement of the long-term survival of RTE by overexpression of CD127 in lymphoreplete or lymphopenic conditions [20, 49, 60–67]. In our cohort, IL-7-depend mechanisms of peripheral T cell renewal may be less predominating, as our YATEC patients showed no lymphopenic situation at the time of evaluation of immune parameters. However, significantly decreased TRECs and shortened telomeres as markers of the replicative history of individual cells indicate for an increased peripheral naive T cell turnover.

In order to assess a possible role of gut-derived or even mucosa-experienced T cells [68], CD103, which is necessary for T cell homing and retention in the gut and other epithelia [69] was included into the analysis. YAT patients showed significantly higher proportions of circulating CD103+ CD8+ T cells. Moreover, despite der relatively young age of YATEC and YAT compared to OHC, proportions of CD103+ CD8+ T cells in thymectomized patients were similar to OHC. Peripheral CD8 + CD103+ memory T cells have been described as non-migratory T cell subpopulations that are maintained as tissue-resident memory T cells without replenishment from the circulating memory T cell pool [29, 70, 71], but CD103+ T cells also contribute to T cells with effector functions [38, 69, 72]. Increased total CD3 + CD103+ numbers were also found in one study investigating children who had thymectomy at least 5 years ago [11] suggesting extra-thymic T cell maturation [27]. We could demonstrate that this effect persists also in our thymectomized patients who had thymectomy more than 25 years ago. The increase of CD103 + CD3+ T cells was previously postulated to reflect extra-thymic T cell generation [27], however, in peripheral blood it is difficult to distinguish between newly generated T cells in the gut versus expansion of previously existing CD8+ memory T cells.

CMV was suggested as a chronic stimulating factor for peripheral T cells [24, 41] and was therefore investigated in thymectomized patients. One hypothesis is that an immune system exposed to the strong pressure by CMV [41] prematurely exhausts its resources in the context of diminished thymic output [17]. A potential risk of early thymectomy was seen for the development of premature immunosenescence and of an immune-risk-phenotype [1, 17, 23] which is defined as a cluster of immune features (e. g., decreased numbers of naive T cells, abundance of highly differentiated memory T cells, increased inflammatory markers, reduction in TCR diversity and CMV seropositivity), which were predictive of early all-cause mortality in one elderly cohort [73]. CMV positive thymectomized patients of our cohort demonstrated lower proportions of CD8 + CD45RA + CD27 + CCR7+ T cells which were independently influenced by CMV and associated with an accelerating effect of CMV on proportional reductions of naive T cells as seen in elderly. However, our results were hampered by low numbers of CMV positive subjects in each group and by the examination of relatively young thymectomized patients which may display less pronounced effects than older ones [17]. In addition, usually the effect of CMV is time-dependent and heterogeneity in age of primary CMV acquisition may vary from study to study.

Considering low thymic output and regenerative peripheral mechanisms, we hypothesized that thymectomized patients may have a restricted TCR repertoire. In fact, a skewed TCR diversity could be found in thymectomized patients. Thus, we could confirm the results of a study investigating the TCR repertoire in YATEC patients compared to young adults and elderly controls which showed mild to strong alterations of the TCR patterns [17]. In that study, strong alterations could be reported in the CD8+ T cell Vβ families, whereas only a few CD4+ T cell Vβ families were affected [17]. The high variability in our thymectomized cohort may be induced by investigating Vβ families in CD3+ T cells without differentiation into CD4+ and CD8+ T cells. However, also at this level and despite small subgroups, changes were evident with a trend towards monoclonal patterns in patients infected with CMV. Variety of results and existence of outliers may result from regeneration of remaining thymic tissue [12, 20]. Additionally, CMV may also not be the sole factor influencing the TCR repertoire in our thymectomized patients [17, 74–76].

Age plays a crucial role in homeostatic mechanisms. All alterations expected in the elderly [26, 42, 44], were found in our OHC control group, such as low proportions of CD31-expressing RTE, increased Ki67 expression of peripheral T cells and higher proportions of CD103-expressing circulating T cells. CD127 expression was lower in OHC and a great variability was found for IL-7 concentrations in OHC, suggesting an inverse relationship between IL-7 receptor signaling function and age [77] with ongoing controversy as to whether IL-7 concentrations are altered with age [77–80]. Despite decreased proportions of CD127-expressing T cells with age [65], higher proliferative activity in CD127+ T cells of OHC was found in our study. This trend was similar between YATEC and OHC regarding proportions of CD127+ in naive CD8+ T cells and the Ki67+ expression in CD127+ memory CD8+ T cells.

## Conclusion

In conclusion, thymectomized patients demonstrated the outstanding situation of artificial depletion of thymic output in an otherwise healthy immune system. We could show that thymectomy is associated with an impairment of the peripheral CD8+ T cell pool. To guarantee renewal of the pre-existing naive T cells, the human immune system seems to struggle in order to fill the void of RTEs after childhood thymectomy by peripheral T cell proliferation, IL-7-mediated mechanisms, and release of CD103+ T cells into circulation. But it cannot avoid skewing of the TCR repertoire and has to deal with chronic viral infections. Despite the moderate changes in T cell proportions, proliferation rates and TCR diversity, no clinical relevant immunodeficiency seems to result from thymectomy in early childhood. However, as many of these findings in adolescents and young adults were reminiscent of immune alterations after thymic involution in the elderly, thymectomized patients may mimic premature immunosenescence [1, 9, 23]. However, not all immunological parameters investigated in thymectomized individuals in this study resemble the findings of elderly persons and in some cases, only trends could be shown. The relatively small immunological alterations found in young adults thymectomized at early infancy or childhood may precede more substantial alterations in later life as suggested by our previous investigations and clinical studies [9, 10, 16, 22, 23]. There is still the possibility that these patients are at risk to suffer from age-related diseases, such as autoimmunity, cancer, atherosclerosis or neurodegeneration, in older age. Also, latent herpes virus infections usually acquired during childhood may have a more pronounced impact on an immune system compensating for thymic loss. Delayed antibody responses to new antigens, such as tick-borne-encephalitis vaccination [22], have suggested that an intact thymus is also necessary for antibody production and affinity maturation [81]. Thus, ongoing follow-up of immunological activity of thymectomized patients is mandatory as they advance into old age to timely recognize age-associated diseases and premature impairments of immune function.
